# Post-Graduates of the Hired Man*From *The Survey*, March 8, 1913.

**Published:** 1913-05

**Authors:** 


### Post-Graduates of the Hired Man.*

In the *School Bulletin,* Prof. C. R. Bardeen, dean of the medical school of the University of Wisconsin, writing on the subject of sex instruction, says:

"Curiosity, passion and idleness are the only features outside of medicine that call attention to sex glands. * * * Public talks to children will be pretty certain to arouse curiosity. They certainly will not subdue passion. In so far as curiosity concerning sex matters is spontaneous and natural, it may best be turned in legitimate directions by quiet private talks with pure-minded friends. * * * So far as passion is concerned, it can be controlled only by the habit

*From *The Survey,* March 8, 1913.

of self-control and the right kind of personal ambition, and these can best be cultivated in the young without reference to the physiology of sex."

This is just such a statement as you would expect to see from the pen of one whose point of view toward sex instruction is purely academic. On the day I read it one of the older boys of the orphanage who had just returned from a ten years' experience in both rural and urban life in Illinois, Kansas and California called to see me. It was a pleasure to look into the ruddy face and clear, blue eyes of a broad-shouldered, deep-chested, 180-pound, husky young man of twenty-four, who had never been sick a day since leaving the home and who bore every evidence of living a clean, pure life. But as I inquired after this one and that one who had left the orphanage at about the same time he was discharged, the pictures he drew of their lives were not all bright.

"Only a few weeks ago," he said, "in Kansas City, I ran across Will F—. He was down and out. Health broken, no money and no work most of the time. Women ruined him; he had been a victim of gonorrhea for six years. I gave him a lift for a couple of weeks, but he's too far gone. I persuaded him to write to his sister while he was with me. He had not heard from her for several years. Of course, he didn't tell her about his loose living. She answered at once, and you should have seen him shed tears when he read the letter, especially where she asked what church he attended. The poor devil hadn't seen the inside of a meeting house in five years.

"But a funny thing happened while he was with me. I was walking with him one day, and all of a sudden he said: 'Yonder is a fellow that looks like my brother. He's in the West somewhere, you know, and I believe that's him.' The young man referred to looked like a sick hobo. He was a little lame, his clothes hung slouchily upon him. We went up to him and when Will asked if his name wasn't F— he said, 'What business is it of yours what my name is?' But when Will gave him his own name and told him he looked like his brother Robert, Rob then recognized him and was glad to see him.

"But holy mackerel! it would have made you sick to look at him. He had been discharged a few days before from the hospital, where he had been operated upon for venereal disease. One leg was shorter than the other, and poor Rob didn't look as if he'd ever be well again."

I remembered those boys well. They were both bright grammar-school pupils, sound and well developed physically, with as promising a future as opens to most American boys. Both went on farms at fourteen years of age. At that age they were ignorant of sex knowledge except such crude information as they may have picked up from other boys. Whatever they learned afterward in that field they gathered, as hitherto practically all farmer boys have done, from vile sources. The lewd talk of the typical hired man! I know all about him. He was my teacher and he was on every farm of considerable size in Illinois in my boyhood days. But he's no worse than the young men of the village, or the city laborer with whom boys are in daily touch, except that his isolated life provides less of other attractions to take up his mind.

It is to such as these, the sewer gateway to sex instruction, that millions of the youth of our country are obliged to go to satisfy that "curiosity concerning sex matters" of which Dean Bardeen speaks. But these teachers don't wait upon the demands of curiosity from their boy pupils. They are ever on the initiative with sex instruction, anxious to impart all they know—and more than they know—upon a vital theme so close to youth as to involve the very life here and destiny hereafter. Their lewd talk is the expression of their own uncontrolled sex emotions, while at the same time it awakens like feelings in their innocent but eager pupils.

"In so far as curiosity concerning sex matters is spontaneous and natural, it may best be turned in legitimate directions by quiet private talks with pure-minded friends."

What a strange view to take of such an important human interest! Can you mention an asset or concern of humanity that is fraught with greater possibilities for good on the one hand or evil on the other than sex physiology and the functioning of the sex organs? Is it reasonable or safe to leave instruction upon a subject so fundamental to social well-being and personal happiness to the accidental suggestions or demands of curiosity? Thousands of the youth of our country are taking steps toward moral ruin every day, like the two boys above mentioned, in almost absolute ignorance of consequences. Girls are as unprotected by the safeguards of proper instruction as boys. But they are more fortunate in their environment.

The greatest mistake we are all making is the assumption that if we, the teachers and parents, are not imparting sex instruction no instruction is being received, whereas the real condition is that the air is full of it. No boy can escape it. The home, the school and the Sunday school have not a monopoly of learning. They cover most fields of knowledge, but here is one which they have not dared to enter. It is closer to the child and more vital to every interest of society than three-fourths of the things we try to teach in these institutions. The child does not wait for this instruction as he does for other learning. It is thrust upon him and he welcomes it. He finds it in the street, in back alleys, behind doors, in secret places, and in the dark. His teachers are the hired man, the teamster, the servant girl, the store clerk, the laborer, the loafer, the barroom habitue and the moving picture show. They have preempted and hitherto held this field of learning for the young. As evidence of this just inspect the walls and partitions of the toilets of almost any public school in the country, or associate for a few days with groups of boys or men anywhere. Our boys cannot escape the obscene signs and drawings or the vile songs and stories which are all about them.

The walls of the toilet room of the high school in which two of my boys graduated were decorated with vulgar markings. They were sandpapered and retouched from time to time by the janitor, only to be defiled again by the procession of new students.

No safeguards are thrown about the child as he receives this instruction from such sources. The low pleasure of lust, with no emphasis upon the danger or responsibility of sexual indulgence is the phase presented to the youthful pupils. Is it any wonder that the two boys whose sad story is briefly told above and thousands like them are going to destruction every day? It would be a greater wonder if they did not do so. They had learned a great deal about the dynamo of human passion without acquiring any knowledge of how to protect themselves from its dangers.

The young, the ignorant and the uncultured, however, have not a monopoly of this sort of thing. Several years ago, when a meeting of the superintendents' association of the National Educational Association was held in Chicago, one of the courtesies of the local committee was an excursion on the lake. A young superintendent, who has since climbed to the top of the educational ladder, now president of one of our large universities and whose home training, religious training, virtuous heredity or some other blessed advantage over most of us, had protected him from or rendered him immune to the contagion of obscene song and story, told me on his return from the meeting how dumfounded he was during this little excursion to hear from the lips of men holding prominent positions as superintendents, principals and professors, jokes, illustrations and stories full of obscene imagery and lewd suggestion. This was about twenty-five years ago. I believe a change for the better in this profession has been going on since that time—a real progress in refinement and culture—and that a similar criticism under like conditions could not be made today. But this improvement at the top has not reached very far down into the substrata of schools, and never will until we take hold of sex instruction in an intelligent and vigorous manner.

The mere fact that in order to teach sex knowledge we must use a refined or scientific vocabulary elevates the subject and dignifies in the minds of the pupils the sex organs and their functions. The vulgar terms, obscene signs and smutty stories which boys habitually hear and associate with these organs degrade their sacred functions and naturally lead the youth to regard the sex parts of our anatomy as vulgar and impure.

There is, as every one knows, a complete unwritten vocabulary of terms for the sex organs and their functions. Thousands of youths all about us grow to manhood without learning or even hearing any other terms for these parts than those of this wretched outcast vocabulary. Is it, therefore, any wonder that even the respectable family man, who loves his home and who has made the most sacred use of the sex organs in bringing sweet and happy children into the world, should still, when alone with men companions, lapse into the loose and low story and rhyme which represent the only sex instruction he ever learned in his youth? These early impressions, vile and catchy as they were, wore the brain grooves too deep to be effaced.

Only a few days ago a young man under twenty, a member of a boat club near my home, returned from a rowing trip with several other men of the club. He expressed disgust at the prevalence of obscene suggestions, filthy jokes and incidents in their conversation on the trip. Several of them were married men. They were only giving expression to the thoughts, vile doggerel and imagery they had habitually associated with sex knowledge from childhood.

Most children learn while still very young that the subject of which we are speaking is tabooed in practically all places where instruction in other subjects is given. This gives it the added attraction of "forbidden fruit," and it is partaken of with all the more zest. Once bring it into the open, give it the same frank and refined treatment that we give other subjects and you will rob it of a large part, if not all, of this unwholesome, hidden, stolen-fruit element of attractiveness. Many a child instructed in this vulgar school of secrecy has been surprised to hear father or mother or teacher actually speak the name of a sex gland as they would any other organ of the body. Usually the only person who ever has the "immodest audacity" to do this is the doctor, and we accord to his profession license to do so. This veto upon the whole subject by decent society is what causes the child to seek his information under cover.

What defense can we offer for such a condition? I believe none whatever except tradition and the mistaken notion that the relation between knowledge and sin is more dangerous than that between ignorance and sin. We greatly fear that the child will "know too much," and therefore meet his innocent questions with all sorts of lies and evasions.

I well remember the sudden advent of a little calf on our place when I was less than six years of age. I asked my mother where the mamma cow got it. In her answer she doubtless followed the example of her mother and told me the cow "found it in the woods." (We had a woodland pasture.) This greatly aroused my imagination, made me as restless and filled my mind with as many more questions as that of the small boy whose inquisitiveness annoyed a cripple. When asked about his misfortune he extracted from the child a promise to ask no more questions, provided the one then pending were answered, namely, how the cripple lost his right foot. "Well, my boy, it was bit off," said he. A friend of mine who was given a similar answer by her grandfather upon the appearance of a litter of pigs told me how she and her little companions tired themselves out hunting the farm over for some more pigs. Such is the satisfaction that many children receive from their innocent inquiries.

You who hesitate to impart sex instruction to children should bear in mind two things—first, that instruction from impure and forbidden sources is certain to reach at least nine-tenths of them without any effort or concern whatever on your part; and, second, that nine-tenths of the danger involved lies on the side of either unwholesome instruction and the secret method by which it is imparted or of ignorance. While you hesitate, boys and girls, young men and women, are going to perdition.

There is another aspect of sex instruction that is peculiar and which puts a compelling force into the demand for it early in the child's life, namely, the exuberant rush of passion which seizes the child in his adolescent period. Toward other fields of knowledge he is comparatively indifferent; toward this he is keenly alert and vehemently responsive. This is why instruction does not wait upon the natural teachers of the child—the parents, or those provided for him in the school or the church. He is too eager; he will take any instruction from any source whatever. The more obscene and suggestive the method the more it arouses his passions, and if he has received no previous instruction—as most boys and girls have not—

he knows none of the dangers and feels little of the moral restraint necessary to safeguard his purity of character. Much of what he learns at this time could have been taught to him earlier before he felt any other than an intellectual or academic relation to such knowledge. If this were done it would go far toward inhibiting his welling up of the sex passions at any or every suggestion, picture or exhibit in nature of animal mating, which many a youth feels and suffers in silence.

